# Risk‐adapted therapy in follicular lymphoma: Is it time to “FLEX”?

**DOI:** 10.1002/ajh.26016

**Published:** 2020-10-24

**Authors:** Joshua W. D. Tobin, Maher K. Gandhi

**Affiliations:** ^1^ Mater Research University of Queensland Brisbane Queensland Australia; ^2^ Princess Alexandra Hospital Brisbane Queensland Australia

Although it remains incurable with conventional chemoimmunotherapy, modern studies estimate the median survival of advanced‐stage follicular lymphoma (ASFL) patients to be approaching 15‐20‐years.^1^ Current clinical prognosticators (typically follicular lymphoma international prognostic index (FLIPI), but also FLIPI‐2 and PRIMA‐prognostic index (PRIMA‐PI)) are useful in categorizing progression free survival (PFS), but poorly identify the 15%‐30% of patients that experience early progression of disease (POD) or histological transformation. With this in mind, Mir and colleagues introduce a new prognostic score for patients with symptomatic ASFL, termed the FL Evaluation Index (FLEX).^2^ The concept is an attractive one, since this sub‐group of patients have poor outcomes with standard treatments and hence represent an unmet need.^3^

So, FLEX incorporates nine dichotomous variables, that provide surrogate information which can be broadly categorized as encompassing patient biological fitness (sex, hemoglobin, performance status), tumor bulk and site (sum of the products of lesion diameters, and number of extranodal sites), biology (lactate dehydrogenase, histological grade, B2M) and immunity (circulating natural killer cell count “NKCC” that reflects the role of NK cells as key effectors of antibody mediated cell death in anti‐CD20 antibody treated patients). The authors were able to demonstrate greater precision in predicting early progression than either FLIPI, FLIPI‐2 or PRIMA‐PI in a training cohort from the large and well‐documented GALLIUM trial, with the greatest separation in bendamustine/anti‐CD20 antibody treated patients. However, the clinical significance of this refinement on prior clinical scores remains uncertain, as strictly speaking, FLIPI (which shares two of its five parameters with FLEX) seems to be broadly equivalent to FLEX within the validation cohort in terms of differential 2‐year and 3‐year PFS. Of note, the validation cohort did not contain bendamustine/anti‐CD20 antibody combinations. It remains to be seen whether any of these clinical prognostic tools retain their utility in the setting of emerging chemotherapy‐free treatment approaches, particularly lenalidomide‐based regimens.

From a pragmatic vantage‐point, prognostic tools must be simple, reproducible, affordable and accessible to real‐world clinical centers in order to be widely embraced. This remains a major reason why combination clinico‐sequencing based scores have not been widely adopted.^4^ While more cumbersome than other clinical risk scores, the wide uptake of on‐line medical calculators should easily facilitate the utility of the FLEX score in practice. The necessary compromise of accuracy and complexity of the model is underscored by the demonstration that the PRIMA‐PI, the most parsimonious of the models described, was least reproducible between the training and validation cohorts.

The FLEX variables are mostly routine in the initial assessment of ASFL and the required techniques are inexpensive and accessible in clinical practice. However as acknowledged by the authors, the CT‐measured “sum product of lesion diameters” is a resource intensive process often reserved for clinical trials. They note that the substitution of “bulky disease” as an alternate measure of disease burden retains acceptable performance of the model, however this data has not yet been presented and demonstration of the validity of this “modified‐FLEX” will be required. Similarly, NKCC is not routine practice in many centres and substitution for this variable, if required, remains to be explored.

Despite the high sensitivity of the FLEX score, not all early POD events were captured. This highlights the significant heterogeneity within this patient group and emphasizes the clinical need for integrated models that also capture unique biological drivers of early progression. In addition to improving on prognostic accuracy, additional molecular measures of disease biology could be leveraged as “predictive biomarkers” to demonstrate differential treatment efficacy between treatment arms.

There are a large number of small molecules and immune based agents at various stages of the translational pipeline. Owing to their non‐overlapping mechanisms of action, there are a variety of ways they can potentially be combined with each other and/or chemoimmunotherapy. Furthermore, as FL is an indolent lymphoma, a long duration and large number of patients are required to conduct these studies. For all these reasons, it is acknowledged that the clinical trial pipeline in ASFL is becoming unmanageable.^5^ Strategies that facilitate a more focused, mechanism based clinical trial program are urgently required. The incorporation of informative biology driven biomarkers along with clinical scores into clinical trial design, is a necessary component to assist in this process (Figure 1). However, there are substantial challenges in developing a biology‐guided strategy to ASFL management. Many past efforts to define predictive biomarkers are conducted retrospectively in non‐uniformly treated cohorts and lack the appropriate study design and power to definitively prove predictiveness for any individual treatment arm. Notably, a correlative study performed on the phase three randomized PRIMA trial (in which rituximab maintenance was evaluated after rituximab plus chemotherapy induction) identified a 23‐gene transcriptomic signature characteristic of B‐cell centroblasts that was prognostic independently of FLIPI.^6^ However, this score had low sensitivity for early POD and was developed prior to adoption of bendamustine/anti‐CD20 antibody therapy.

**FIGURE 1:**
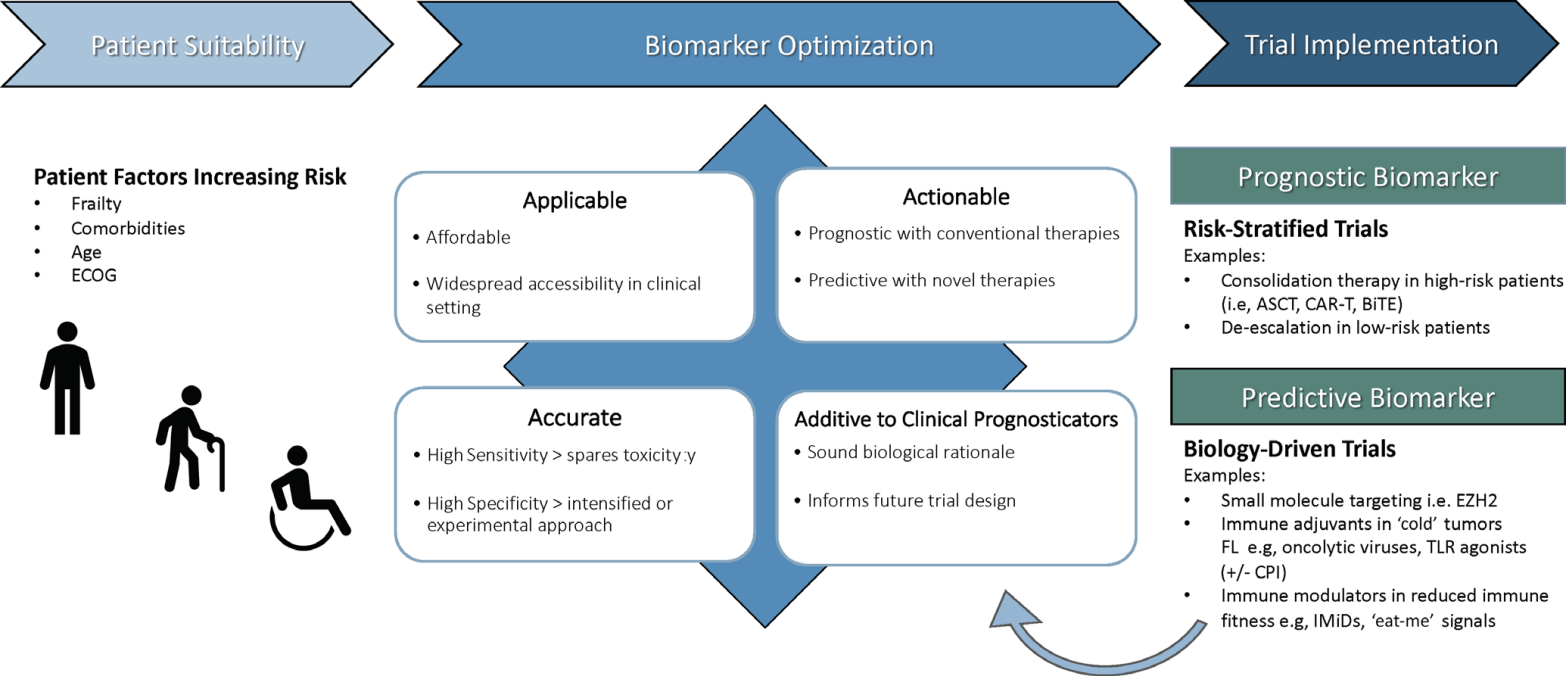
Conceptual framework for the synchronous development of therapeutic and biomarker pipelines. ASCT, autologous stem cell transplantation; TLR, Toll‐like receptor; BiTE, Bi‐specific T‐cell engager; CAR‐T, chimeric antigen receptor T cell; CPI, checkpoint inhibitor; ECOG, eastern cooperative oncology group performance status; EZH2, enhancer of zeste homolog 2; IMiDs, immunomodulatory drugs; TLR: Toll‐like receptor

In the age of precision medicine, the ideal biomarker must also be underpinned by strong basic science. A sound biological rationale will far more likely gain acceptance amongst the hematology community, and in turn this is more likely to lead to its widespread adoption. Importantly, recent studies have identified characteristics that promote or circumvent vulnerability to specific agents in FL. This is best shown by the results of the EZH2 inhibitor tazemetostat, which demonstrates differential response rate in those carrying a mutated vs wild‐type EZH2 gene.^7^ These findings underscore the need for a biologically driven definition of patient populations that may benefit from a specific treatment with molecularly targeted agents. In addition, oncogenic pathways in malignant cells also strongly influence the nature of the anti‐tumor immune response.^8^ The relationship between the extent of immune infiltration within the tumor microenvironment and patient outcome has recently been identified.^9^ Patients with FL tumors characterized by robust transcriptional upregulation of immune genes, numerous macrophages and clonal CD8^+^ T cell expansions (ie, inflamed or “hot”) were substantially less likely to experience early POD. By contrast, patients with a microenvironment largely devoid of infiltrating immune cells (ie, ” cold”) were enriched in early progressors. Potentially, in this scenario agents that rectify a cold microenvironment by induction of an inflammatory response (eg, radiation, oncolytic viruses, TLR‐agonists) might be beneficial.

In summary, the FLEX score is an easily applicable and accurate clinical prognostic tool that may outperform prevailing prognostic models for predicting early adverse events, particularly in those that receive a bendamustine backbone. This positions the FLEX as an ideal “prognostic backbone” upon which genetic and immune biomarkers, perhaps in combination with dynamic markers such as PET‐scans and cell‐free DNA,^10^ could be added to further improve the models prognostic and predictive accuracy, as well as being central to the development of rational biomarker‐driven trials.

## CONFLICT OF INTEREST

None.

## Data Availability

Not applicable.
